# Impact of vitamin D supplementation in the prognosis of patients with SARS-CoV2 pneumonia admitted to the intensive care unit – a randomized controlled trial

**DOI:** 10.3389/fimmu.2025.1593200

**Published:** 2025-06-05

**Authors:** Ana Moura Gonçalves, Bárbara Sucena Rodrigues, Maria Lobo Antunes, João Gonçalves, António Marinho

**Affiliations:** ^1^ Department of Intensive Care Medicine, Hospital Beatriz Ângelo, Loures, Portugal; ^2^ Faculty of Pharmacy, University of Lisbon iMed – Research Institute of Medicines, Lisbon, Portugal; ^3^ School of Medicine and Biomedical Sciences, Instituto de Ciências Biomédicas Abel Salazar, University of Porto, Porto, Portugal

**Keywords:** SARS-CoV-2 pneumonia, COVID 19, vitamin D, cholecalciferol, critical care patients, polyvalent intensive care unit

## Abstract

**Background:**

Research suggests that patients with low vitD levels are more susceptible to severe SARS-CoV-2 infections with need for hospitalization and admission to an intensive care unit (ICU). Our objectives were to evaluate the impact of cholecalciferol supplementation in critical care patients with severe SARS-CoV-2 pneumonia in regards to prognosis, evolution of organ failure and need for organ support.

**Methods:**

A nonblinded controlled trial was conducted in patients with severe SARS-CoV-2 pneumonia admitted to the ICU. Patients were randomized by block of two, into three groups (no cholecalciferol, 500MU cholecalciferol arm, 2MU/day cholecalciferol during ICU stay and remaining hospitalization). Serum 25-hydroxyvitamin D levels were measured and correlated with organ failure indicators (based on SOFA), ICU length of stay, need for organ support, days on mechanical ventilation and ICU, intra-hospital and 60 day mortality.

**Results:**

207 patients were included. The number of organ failures showed a significant negative correlation with 25vitD levels on admission (r= -0.208, p=0.005), on the third day (r= -0.312, p<0,001), and on the seventh day(r= -0.224, p=0.01). In the group of patients supplemented with 500MU of cholecalciferol there was a significant negative correlation between the number of organ failures and 25vitD levels on third day (r= -0.454, p<0.001).

**Conclusions:**

Lower vitD levels on admission were related to more organ failures and high doses cholecalciferol supplementation was related to lower organ failures. More studies are needed to evaluate the impact of baseline vitD levels on clinical outcome and prognosis, to identify potential subpopulations that can benefit from supplementation and to understand the impact of critical illness on cholecalciferol action and protective effect.

## 1 Introduction

Low serum levels of vitD are a global concern, impacting over a billion individuals worldwide (1, 2).

The serum level of 25-hydroxyvitamin D (25vitD), a precursor to 1,25 dihydroxy vitamin D (1.25vitD), is the primary indicator of vitD status and has a long half-life of 25 days. 1.25vitD is dependent on parathyroid hormone and renal hydroxilase activity, and its levels can be normal until a very severe decrease in 25vitD levels (3, 4). There is ongoing debate about reference values and cut-off points for vitD levels (5, 6). Some studies suggest that serum levels of 25vitD above 75 nmol/L (30 ng/mL) are beneficial, with an optimal range between 90–100 nmol/L (7, 8). Other studies define reference levels for 25vitD between 30–80 ng/mL (75–200 nmol/L), with concentrations below 10 ng/mL considered severely low (9, 10). A seasonal variation in vitD levels is well-documented (11) and has been linked to seasonal fluctuations in influenza and respiratory tract infections (12). Lower vitamin D levels in winter may contribute to seasonal illnesses such as influenza (13, 14). The relationship between vitamin D levels and previous UV exposure remains unclear (15).

Studies have shown that low levels of vitD may increase the risk of infectious and autoimmune diseases (16–19). In addition to its role in calcium homeostasis, vitamin D may also play a role in regulating inflammation and protecting against viral and bacterial infections (20, 21).

Low vitamin D levels have been linked to increased rates of respiratory tract infections in both children and adults (22, 23) and are associated with poor prognosis, longer disease duration, increased complications, and higher mortality rates (24, 25).

Deficiency in vitamin D is common among critically ill patients, with prevalence ranging from 40 to 70% (26–28). This deficiency may be due to factors like drug interactions, fluid resuscitation, malnutrition or metabolic dysregulation, leading to decreased vitamin D levels upon ICU admission (29–31). Multiple studies have reported decreased vitamin D levels in critically ill patients and the association with conditions such as sepsis, acute respiratory distress syndrome, and acute kidney injury (32–34), need for mechanical ventilation and/or even with higher mortality (35). Some authors consider vitamin D equivalent to a negative acute-phase protein, leading to low serum levels during acute systemic inflammation (36). In septic patients, vitamin D appears to play a vital role in host defense (37), with a known association between lower levels and worse ICU prognosis (38). Lower serum levels of 25vitD are associated with an increased risk of COVID-19 infection (39–41). In COVID-19 patients, vitamin D levels were found to be reduced, with lower levels correlating with a poorer prognosis (42, 43). Deficiency of 25vitD was associated with a more pronounced systemic inflammatory response, an increased risk of respiratory failure (44), need for mechanical ventilation and even increased death (45). During the COVID-19 pandemic, the role of vitamin D in protection against the virus and the efficacy of supplementation came under scrutiny. While some studies found no direct link between vitamin D levels and COVID-19 mortality (46), others reported lower vitamin D levels in COVID-19 patients compared to controls, suggesting a potential association with disease severity (47).

Vitamin D supplementation has been associated with the prevention of respiratory tract infections for years (48). It has been shown to enhance the production of cathelicidin antimicrobial peptide, which is crucial for defense against bacteria, viruses, and fungi (49–53).

In critical care patients the efficacy of vitamin D supplementation remains inconclusive, perhaps because of the heterogeneity of patients. Some studies suggest that it may reduce the duration of mechanical ventilation and ICU stay, whereas other research has failed to demonstrate significant improvements in mortality or infection rates (53, 54). Interestingly, some studies suggest that rapidly restoring vitamin D levels in critically ill patients with severe deficiency could prevent viral respiratory infections or reduce mortality rates (55–57). Caution is advised, as high doses of vitamin D may lead to adverse effects such as hypercalcemia, hypercalciuria or hypervitaminosis D particularly in pediatric patients (58, 59) and poor formulated or unlicensed vitamin D can be deleterious (60). However, there is evidence that high dose of vitamin D is safe (61, 62), with mild hypercalcemia being a rare adverse effect in adults (63). In critically ill patients, high doses of vitamin D were given without reported adverse effects (57, 64).

Studies on vitamin D supplementation in critically ill COVID-19 patients have yielded conflicting results (65–67). Given the ongoing research and heterogenous results, further studies are needed to determine the optimal dosages and potential benefits of vitamin D supplementation in critically ill patients with severe COVID-19 pneumonia.

## 2 Materials and methods

### 2.1 Selection of patients and study design

This one center study included critically ill patients admitted to a polyvalent intensive care unit during a period of one year (November 2020 to December 2021) for severe SARS-CoV-2 pneumonia, with or without associated ARDS. Patients could have other comorbidities as long as their decompensation was not the reason of ICU admission.

Severe COVID-19 pneumonia was defined as respiratory failure caused by SARS-CoV-2 lung infection causing ICU admission for non-invasive and/or mechanical ventilation. See severe pneumonia definition below.

Three critically ill patients randomized groups were established (randomized by blocks of two, according to the order of entry into the ICU, without prior knowledge of the allocation key) to whom cholecalciferol was given or not in moderate or high doses (nonblinded, randomized, controlled study).

The ICU admission of the patient was a responsibility of senior Intensivists, without knowledge of the allocation key.

Patients were placed into the study, sequentially: 2 patients in the cholecalciferol-free arm. Next 2 patients in the cholecalciferol 250 000U/day arm (12.5 mL of 0.5 mg/ml cholecalciferol oral solution, orally or by nasogastric tube) for 2 days, and the next 2 patients in the 2000U/day cholecalciferol arm (3 drops of 0.5 mg/ml cholecalciferol oral solution, orally or by nasogastric tube) during the ICU stay and remaining hospitalization.

This allocation was made by the physicians responsible for patient admission, after consulting the allocation arm.

Serum 25-hydroxyvitamin D (25vitD) levels were measured on admission and after, according to protocol (Supplement 1), and correlated with organ failure indicators (based on SOFA), ICU length of stay, need for organ support, days on mechanical ventilation and ICU, intra-hospital and 60 day mortality.

Exclusion criteria were: patients aged under 18 years, pregnant women, patients with an estimated ICU or hospital stay of less than 48 hours, surgical patients, patients with prolonged decompensated heart failure, patients with shock caused by a condition other than respiratory infection, patients with known stage 4 chronic kidney disease, patients with severe hypercalcemia, with advanced stage neoplasms or other chronic pathologies in progress, namely hematological, patients with no possibility of early enteral feeding and patients supplemented with vitamin D.

The protocol (available in Supplement1) and consent forms had been previously approved by the hospital ethics committee (N/Ref.3362/2020_MJHNO; Study number 495_LH number 180, approved date: 2020-10-02). Written informed consent or deferred consent was obtained from all patients or their legal surrogate.

Clinical evolution, adverse effects and prognosis of patients infected with Covid 19, depending on the presence or absence of therapy with cholecalciferol were evaluated. The main comorbidities of patients, namely arterial hypertension, obesity, diabetes mellitus, among others, were studied as well as concomitant therapies with immunomodulatory effect (corticosteroids and other immunomodulatory therapy), to exclude any relationship with clinical course.

### 2.2 Blood sample analysis

Quantitative determination of 25vitD was obtained by competitive immunoassay, Atellica IM Analyzer, SIEMENS healthineers, Plasma levels of 1,25vitD were analyzed by chemiluminescent immunoassay (CLIA), LIASON XL, DiaSorin Inc; prealbumin was analyzed by nephelometry; albumin by colorimetry; C- Reactive protein and transferrin by immunoturbidimetry and ferritin by chemiluminescence.

### 2.3 Severe SARS - CoV2 pneumonia definition

Patients with SARS - CoV2 pneumonia should have 3 of: respiratory rate >30/min, or PaO2/FiO2 ratio ≤ 250, bilateral infiltrates, confusion/disorientation, hypotermia, hypotension requiring agressive fluid ressuscitation; or 1 of: need for invasive/non-invasive mechanical ventilation or septic shock.

### 2.4 Organ failure definitions

Organ failure was evaluated using a SOFA score adaptation (0- no failure; 1- organ failure):

For hemodynamic failure: need for vasopressor (0 no vasopressor, 1 vasopressor).

For respiratory failure; PaO2/FiO2 (0 >200, 1 <200 or need for mechanical ventilatory support).

For hematological failure (0 platelets > 100x103/mm3, 1 platelets < 100x103/mm3).

For renal failure (0 creatinine < 2 mg/dL, 1 creatinine > 2 mg/dL and/or dialysis needs.

For liver failure: total bilirubin (0 total bilirrubin < 2 mg/dL, total bilirrubin > 2 mg/dL or INR >2.

Total failing organ score was obtained from the sum of failures (0- no failure; 1- organ failure), based on SOFA (above).

### 2.5 Statistical analysis

The distribution of the data was tested with the Kolmogorov–Smirnov test for normality. Results were presented as means and standard deviations if normally distributed or as medians and interquartile ranges (IQR) if non-normally distributed. Categorical variables were presented as frequencies and percentages. For comparisons between categorical variables, the chi-squared test and the Fisher’s exact test were used. For comparisons between numeric variables and categorical variables, we used the two-samples unpaired Wilcoxon test (to compare 2 groups) and Kruskal-Wallis test (to compare more than 2 groups). A p-value < 0.05 was considered statistically significant. When a statistically significant difference was found, we performed a binomial logistical regression analysis and reported odds ratio (OR) with 95% confidence intervals.

A binomial logistic regression to find the risk factors independently related to mortality, and a multivariate logistic regression of mortality outcomes adjusted for the previously determined independent variables, were performed. To analyze ventilation time in three groups and see whether mortality decreased with any of them, we used a survival analysis, such as the Kaplan-Meier analysis, followed by a survival curves comparison test, such as the log-rank test or the Mantel-Haenszel test. All statistical analyses were performed with SPSS version 26.0 (SPSS Inc. Chicago, IL) and RStudio Team (2022). (RStudio: Integrated Development Environment for R. RStudio, PBC, Boston, MA URL http://www.rstudio.com/).

## 3 Results

From the initial 233 patients, 207 patients admitted to ICU with severe SARS-CoV-2 pneumonia were included: 66 patients received 500MU (250MU/day, 2 days) of cholecalciferol on the first 48h, 72 received 2MU of cholecalciferol/day during hospital stay and 69 patients received no supplementary cholecalciferol. 14 patients were not eligible (4 had bacterial pneumonia, 3 died within first 24h, 2 were transferred to other hospital before 48h, 2 were transferred to the ward before 48h and 3 received recent vitamin D therapy).

The demographic characteristics of patients included in the study are shown in **Table 1**. The median age was 57 years and the majority of patients were Caucasian (87.4%) and male (70,5%). Comorbidities were similar between groups, with no statistical significance.

**Table 1:** Baseline characteristics of patients.

Variable	Total (n=207)	500MU Vitamin D (n= 66)	3MU/day Vitamin D (n= 72)	No Vitamin D supplemented patients (n= 69)	p-value
Age - years (sd)	57.7 (13.5)	56.9 (12.6)	57.5 (14.1)	58.6 (13.6)	0.752
Female sex – n (%)	61(29.5)	16 (24.2)	20 (27.8)	25 (36.2)	0.289
Race – n (%) Black White Asian	20 (9.7) 181 (87.4) 6 (2.9)	6 (2.9) 58(28.0) 2 (1.0)	9 (4.3) 41 (75.9) 1 (0.5)	5 (2.4) 61(29.5) 3 (1.4)	0.711
Comorbidities – n (%) Hypertension Diabetes Dyslipidemia Obesity Heart failure Chronic kidney disease COPD	113 (54.6) 60 (29.0) 51 (29.7) 85 (41.1) 15 (7.2) 11 (5.3) 15 (7.2)	31 (15.0) 18 (8.7) 18 (10.5) 27 (13.0) 4 (1.9) 2 (1.0) 8 (3.9)	42 (20.3) 19 (9.2) 14 (8.1) 24 (11.6) 6 (2.9) 2 (1.0) 5 (2.4)	40 (19.3) 23 (11.1) 19 (11.0) 34 (16.4) 5 (2.4) 7 (3.4) 2 (1.0)	0.321 0.618 0.583 0.157 0.876 0.09 0.117
Body mass index - mean (SD)	29.0 (5.40)	29.35 (5.48)	28.46 (5.38)	29.25 (5.41)	0.565
Remdesivir use – n (%)	32 (15.5)	10 (15.2)	11 (15.3)	11 (15.9)	0.991
Corticoid use – n (%) Dexametasone Metilprednisolone	207 (100%) 201 (97.1) 15 (7.2)	66 (100) 66 (100) 3 (4.5)	72 (100) 69 (95.8) 9 (12.5)	68 (98.6) 66 (95.7) 3 (1.4)	0.235 0.104
SOFA score - median (mín-max)	6 (2–12)	4 (2–12)	6 (3–11)	6 (3–12)	0.61
APACHE II score - mean (SD)	10.3 (5.97)	9.47 (4.52)	10.88 (5.83)	10.57 (4.33)	0.225
Risk Mortality APACHE II- median (IQR)	11.33(8.91)	11.33 (7.38)	11.33(10.65)	12.89 (8.53)	0.058
SAPS II score - mean (SD)	33.1(11.73)	31.65(11.03)	33.90(12.67)	33.64 (11.40)	0.477
Risk Mortality SAPS II- median (IQR)	15.29(17.53)	12.80(18.37)	15.30(18.97)	15.30 (17.82)	0.399

COPD, chronic obstructive pulmonary disease; HIV, human immunodeficiency virus; IQR, interquartile range; Mín, minimum, Max, maximum; APACHE, Acute Physiology and Chronic Health Evaluation; SAPS, Simplified Acute Physiology Score; SD, Standard Deviation; SOFA, Sequential Organ Failure Assessment.

Mortality predicting scores, such as SOFA, SAPS II and APACHE II were similar between groups, with a relatively low risk (median SOFA < 6, max 12).

There was no correlation between 25 hydroxyvitamin D (25vitD) levels and ICU (r = 2.313, p = 0.940) or hospital (r = 0.051, p = 0.502) length of stay. However, the number of organ failures showed a significant negative correlation with 25vitD levels on admission (r= -0.208, p=0.005), on the third (r= -0.312, p<0,001), and on the seventh days (r= -0.224, p=0.01).

In the group of patients supplemented with 500MU of cholecalciferol, there was a significant negative correlation between the number of organ failures and 25vitD levels on third day (r= -0.454, p<0.001), but not on seventh day. In patients without any supplementation of cholecalciferol a significant negative correlation was found between the number of organ failures and 25vitD levels on seventh day (r = -0.407, p=0.008), but not on third day.

In the group of patients supplemented with daily dose of 2MU of cholecalciferol there was no significant correlation between the number of organ failures and 25vitD levels on third and seventh days although there was a tendency for less organ failures in patients with higher 25vitD concentrations on third day (r= -0.234, p=0.067).

Despite a 25vitD median level on admission of 15.7 ng/mL without statistically significant difference between groups, there was a tendency towards higher values in the group not supplemented with cholecalciferol (16.6 ng/mL). Other laboratory parameters on admission were also similar between groups (**Table 2**).

**Table 2:** Laboratory parameters at admission (D0), third day (D3) and 7th day (D7).

Variable	Total (n=207)	500MU Vitamin D (n= 66)	3MU/day Vitamin D (n= 72)	No Vitamin D supplemented patients (n= 69)	p-value^*^
Calcium- phosphorus metabolism (D0) - median (IQR) Vitamin D (ng/mL) Total calcium (mg/dL) Phosphorus (mg/dL) PTH (pg/mL)	15.7 (12.0) 8.4 (0.5) 3.5 (1.3) 45.9 (44.0)	15.4 (12.1) 8.3 (0.6) 3.4 (1.2) 48.2 (46.2)	15.5 (11.9) 8.3 (0.55) 3.5 (1.2) 43.0 (51.3)	16.6 (12.8) 8.3 (0.6) 3.5 (1.5) 49.6 (37.7)	0.385 0.957 0.867 0.617
Calcium- phosphorus metabolism (D3) - median (IQR) Vitamin D (ng/mL) Total calcium (mg/dL) Phosphorus (mg/dL) Parathyroid hormone (pg/mL)	14.2 (10.2) 8.4 (0.5) 3.6 (1.1) 37.6 (32.3)	16.6 (14.6) 8.4 (0.6) 3.5 (1.1) 37.6 (31.85)	13.8 (10.8) 8.5 (0.6) 3.8 (1.1) 34.6 (28.9)	13.9 (8.2) 8.4 (0.4) 3.5 (1.0) 42.1 (35.1)	**0.008** 0.666 0.575 0.370
Calcium- phosphorus metabolism (D7) - median (IQR) Vitamin D (ng/mL) Total calcium (mg/dL) Phosphorus (mg/dL) Parathyroid hormone (pg/mL)	14.3 (10.1) 8.5 (0.7) 3.5 (1.1) 34.2 (32.7)	18.2 (14.5) 8.6 (0.7) 3.7 (0.98) 29.6 (24.1)	12.9 (9.0) 8.5 (0.6) 3.6 (1.2) 35.6 (31.9)	12.4 (7.8) 8.4 (0.7) 3.1(1.1) 46.2 (34.5)	**<0.001** 0.281 **<0.001** **0.032**
Inflammatory markers (D0): Positive acute phase proteins- median (IQR): C-reactive protein (mg/dL) Ferritin (pmol/L) Fibrinogen Negative acute phase proteins- median (IQR): Albumin (g/dL) Pre-albumin Other markers - median (IQR): Platelets (x10³/mm³) Creatinine (mg/dL) Total bilirrubin (mg/dL)	9.1 (10,8) 1461 (1542) 581 (203) 3.2 (0.5) 15 (7.2) 225 (126) 0.8 (0.4) 0.5 (0.2)	10.3 (10.6) 1503 (1510) 606 (212) 3.3 (0.5) 15.0 (8.7) 218 (145) 0.9 (0.4) 0.5 (0.2)	9.1 (9.5) 1665 (2304) 568 (150) 3.2 (0.6) 15.0 (9.5) 226 (104) 0.9 (0.4) 0.5 (0.4)	8.6 (12.3) 1205 (1107) 668 (223) 3.2 (0.5) 15.0 (6.9) 230 (164) 0.9 (0.4) 0.4 (0.2)	0.891 0.175 0.827 0.590 0.978 0.967 0.857 0.207
Inflammatory markers (D3): Positive acute phase proteins- median (IQR): C-reactive protein (mg/dL) Ferritin (pmol/L) Fibrinogen Negative acute phase proteins- median (IQR): Albumin (g/dL) Pre-albumin Other markers - median (IQR): Platelets (x10³/mm³) Creatinine (mg/dL) Total bilirrubin (mg/dL)	5.2 (10,0) 1664 (1376) 525 (205) 2.9 (0.6) 15.0 (7.2) 279 (133) 0.7 (0.3) 0.6 (0.3)	5.2 (8.6) 1100 (1026) 511 (199) 2.9 (0.6) 15.0 (8.7) 276 (166) 0.8 (0.3) 0.4 (0.2)	6.2 (11.4) 1371 (1689) 544 (231) 2.9 (0.5) 15.0 (8.5) 282 (118) 0.7 (0.3) 0.4 (0.3)	4.4 (9.8) 1234 (1576) 538 (169) 3.0 (0.5) 15.0 (6.9) 272 (126) 0.7 (0.3) 0.4 (0.3)	0.632 0.153 0.479 0.625 0.801 0.963 0.793 0.650
Inflammatory markers (D7): Positive acute phase proteins- median (IQR): C-reactive protein (mg/dL) Ferritin (pmol/L) Fibrinogen Negative acute phase proteins- median (IQR): Albumin (g/dL) Pre-albumin Other markers - median (IQR): Platelets (x10³/mm³) Creatinine (mg/dL) Total bilirrubin (mg/dL)	3.9 (10,2) 1186 (1120) 554 (266) 2.7 (0.8) 22.8 (12.5) 308 (160) 0.7 (0.3) 0.5 (0.3)	2.7 (10.8) 1168 (866) 574 (312) 2.6 (0.7) 23.7 (10.9) 324 (150) 0.8 (0.3) 0.4 (0.3)	3.3 (9.8) 1237 (1299) 498 (229) 2.7 (0.8) 22.2 (13.2) 317 (176) 0.7 (0.4) 0.5 (0.4)	4.4 (12.9) 1201 (1084) 602 (284) 3.0 (0.7) 21.8 (12.6) 302 (164) 0.7 (0.3) 0.5 (0.2)	0.261 0.665 0.570 0.499 0.740 0.608 0.615 0.804

* Kruskal-Wallis test; IQR, interquartile range.

When comparing inflammatory markers from admission to D7, there were no significant differences between the groups supplemented or not with cholecalciferol (**Table 2**). No association was found between cholecalciferol administration and acute positive and negative inflammatory markers (**Table 2**).

25vitD levels before and after supplementation are presented in **Table 3**. We observed a statistically significant difference between groups on D3 (p = 0.008) and D7 (p <0.001), but not before supplementation (on admission), with higher levels in the 500MU supplemented group at D3 (Z = 1047, p < 0.001) and D7 (Z = 781, p < 0.001). The other groups, including the group supplemented with 2MU/day, decreased serum concentration of 25vitD over time.

**Table 3:** Variation in 25vitD levels during ICU stay.

Variation in 25vith levels^*^	Variable Difference in 25vith levels^*^ between days:	Median difference (ng/mL) with (95%CI)	Total (n=207)	500MU Vitamin D (n= 66)	3MU/day Vitamin D (n= 72)	No Vitamin D supplemented patients (n= 69)
D7 - D1 Wilcoxon (Z) ^*^ p-value	-0.8 (-1.95 to 0.5)	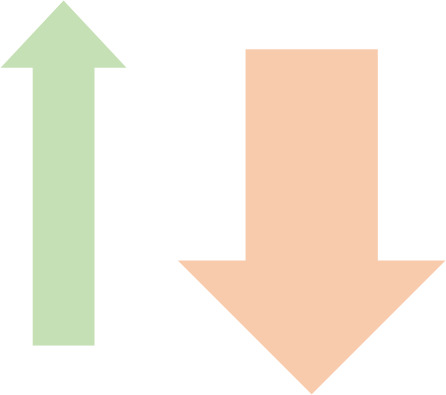 Z= 3462.5 p = 0.661	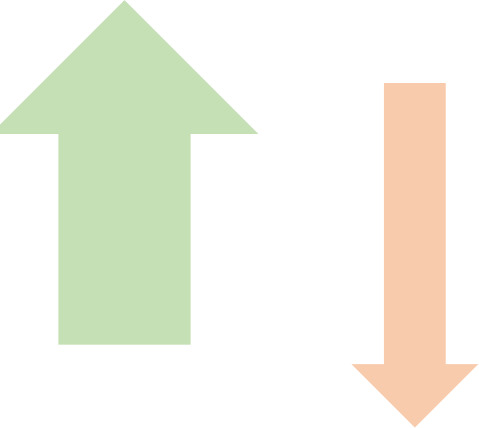 Z= 737.7 **p = 0.005**	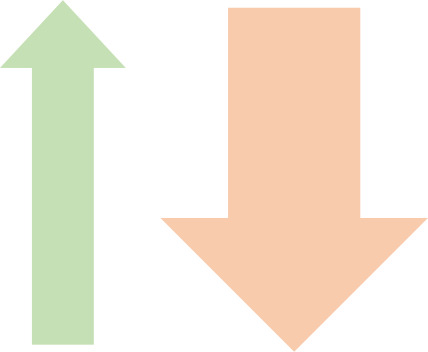 Z= 299.5 p = 0.137	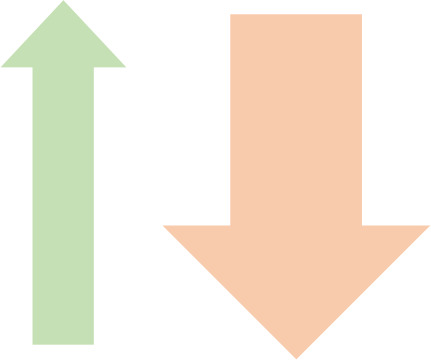 Z= 573.0 **p < 0.001**
D3 - D1 Wilcoxon (Z) ^*^ p-value	-0.35 (-0.9 to 0.45)	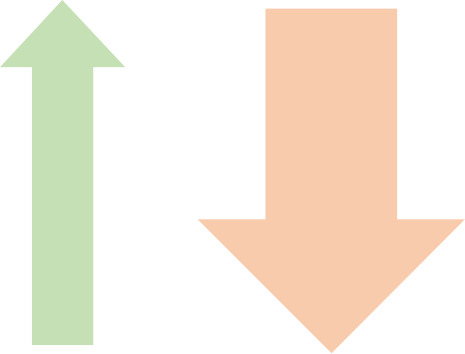 Z= 6272.5 p = 0.684	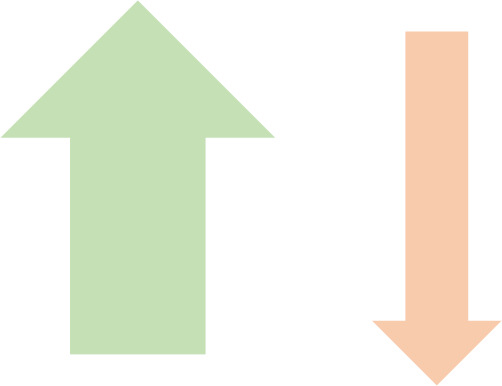 Z= 1047.0 **p < 0.001**	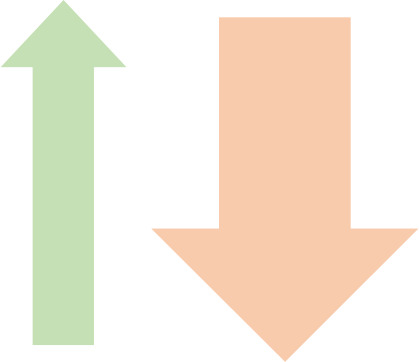 Z= 675.0 p = 0.955	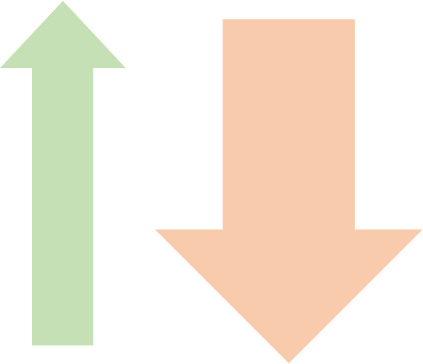 Z= 277.0 **p < 0.001**
D7 - D3 Wilcoxon (Z) ^*^ p-value	-0.1 (-0.6 to 0.7)	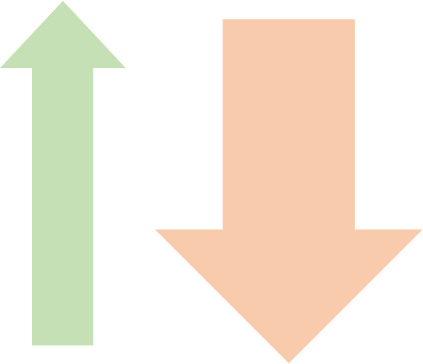 Z = 4471.5 p = 0.327	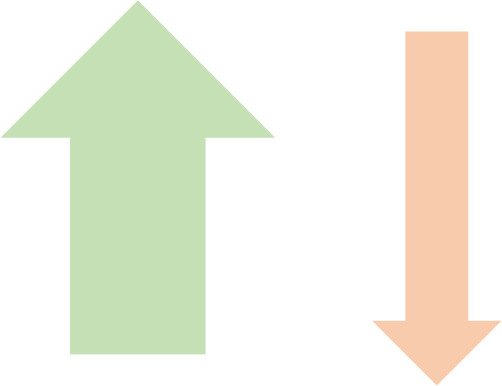 Z= 781.0 **p < 0.001**	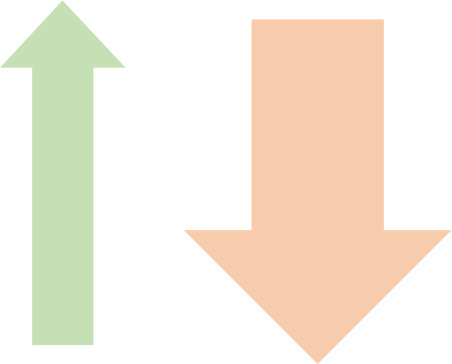 Z= 389.5 p = 0.595	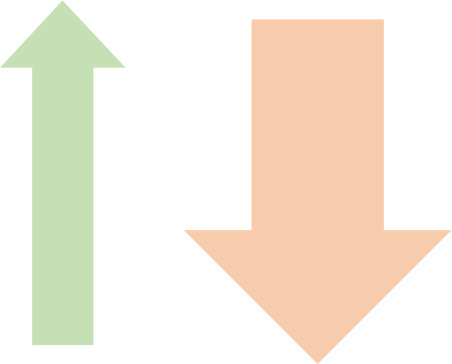 Z= 264.5 **p = 0.019**

* Wilcoxon Signed Rank Test, with selection of cases.

Thin arrows: non-significant/Thick arrows: significant.

Upward arrow: increase in 25vitD levels/Downward arrow: decrease in 25vitD levels.

Although PTH and phosphorus decreased their levels significantly by D7 only in the 500MU supplemented group (p = 0.032 and p < 0.001, respectively), serum levels of total calcium remained similar in all groups (**Table 2**).

Patients admitted to the ICU required some kind of ventilatory support, invasive or noninvasive (NIV) for a median duration of 4 days. Non-invasive ventilation was used in 75.4% of patients and high flow nasal cannula (HFNC) in 38.2%, with invasive mechanical ventilation in 58% (**Table 4**). Vasopressors were used in 44.4% of patients for a median of less than a day, with a maximum of 17 days. Noradrenalin was preferred.

**Table 4:** Organ support therapy during ICU.

Variable	Total (n=207)	500MU Vitamin D (n= 66)	3MU/day Vitamin D (n= 72)	No Vitamin D supplemented patients (n= 69)	p-value
Vasopressor support Need for vasopressors – n (%) Noradrenalin Dobutamin Duration (days) - median (IQR; min-max)	92 (44.4) 87 (42.0) 7 (3.4) 0 (3, 0 – 17)	26 (39.4) 25 (37.9) 1 (1.5) 0 (2, 0 – 16)	32 (44.4) 32 (44.4) 2 (2.8) 0 (3.75; 0 - 13)	34 (49.3) 30 (43.5) 4 (5.8) 0 (3, 0–17)	0.513 0.705 0.365
Ventilatory support Type – n (%) NIV HFNC IMV (with or without NIV) Duration (days) - median (IQR) Curarization – n (%) Prone position – n (%) Extubation failure – n (%) Pneumothorax/pneumomediastin - n (%) Thraqueostomy - n (%)	156 (75.4) 79 (38.2) 120 (58.0) 4.0 (10.0) 51(24.6) 106 (51.2) 24 (11.6) 15 (7.2) 20 (9.7)	54 (81.8) 25 (37.9) 34 (51.5) 3 (9.2) 15 (22.7) 29 (43.9) 9 (13.6) 4 (6.1) 6 (9.1)	54 (75.0) 29 (40.3) 44 (61.1) 4.5 (13.0) 18 (25.0) 40 (55.6) 10 (13.9) 7 (9.7) 10 (13.9)	48 (69.6) 25 (36.2) 42 (60.9) 4 (10.0) 18 (26.1) 37 (53.6) 5 (7.2) 4 (5.8) 4 (5.8)	0.255 0.883 0.436 0.774 0.899 0.350 0.392 0.603 0.262
Acute kidney injury - n (%) Renal support Need for RRT – n (%) Type CVVHDF DRRT	26 (12.6) 18 (8.7) 17 (8.2) 6 (2.9)	10 (15.2) 7 (10.6) 6 (9.1) 2 (3.0)	6 (8.3) 5 (6.9) 5 (6.9) 3 (4.2)	10 (14.5) 6 (8.7) 6 (8.7) 1 (1.4)	0.405 0.747 0.886 0.628
Nutrition – n (%) Oral Enteral Parenteral Intolerance – n (%) Duration (days) - median (min-max)	108 (52.2) 122 (58.9) 8 (3.9) 43 (20.8) 0 (0–16)	35 (53.0) 35 (53.0) 3 (4.5) 13 (19.7) 0 (0–16)	39 (54.2) 45 (62.5) 2 (2.8) 15 (20.8) 0 (0–11)	34 (49.3) 42 (60.9) 3 (4.3) 15 (7.2) 0 (0–16)	0.833 0.488 0.838 0.958
Infectious Complications – n (%) No Tracheobronchitis Bloodstream VAP Bloodstream and VAP Urinary Endocarditis	143 (69.1) 7 (3.4) 14 (6.8) 31 (15.0) 8 (3.9) 3 (1.4) 1 (0.5)	47 (71.2) 5 (7.6) 4 (6.1) 6 (9.1) 4 (6.1) 0 (0.0) 0 (0.0)	49 (68.1) 2 (2.8) 5 (6.9) 11 (15.3) 3 (4.2) 2 (2.8) 0 (0.0)	47 (68.1) 0 (0.0) 5 (7.2) 14 (20.3) 1 (1.4) 1 (1.4) 1 (1.4)	0.286
Therapy used – n (%) Remdesivir Dexametasone Metylprednisolone Profilactic enoxaparin Therapeutic enoxaparin	32 (15.5) 201 (97.1) 15 (7.2) 139 (67.1) 88 (42.5)	10 (15.2) 66 (100.0) 3 (4.5) 43 (65.2) 30 (45.5)	11 (15.3) 69 (95.8) 9 (12.5) 47 (65.3) 32 (44.4)	11 (15.9) 66 (95.7) 3 (4.3) 49 (71.0) 26 (37.7)	0.991 0.235 0.104 0.704 0.606

Missing values were omitted from the analysis.

CVVHDF, Continuous Venovenous Hemodiafiltration; DRRT, Discontinuous Renal Replacement Therapy; IQR, Interquartile Range; NIV, Noninvasive Ventilation; IMV, Invasive Mechanical Ventilation; HFNC, High Flow Nasal Cannula; RRT, Renal Replacement Therapy.

There was no association between cholecalciferol supplementation and time on ventilator (H(2) = 0.774, p = 0.679), vasopressor needs (c2(2) = 1.334, p = 0.513) or renal replacement therapy (c2(2) = 0.581, p = 0.748). Overall, 12.6% of patients suffered from acute kidney injury, and 8.7% needed renal replacement therapy. The number of patients with acute renal failure or acute on chronic renal failure was similar in the 3 groups of 25vitD (c2(2) = 1.809, p = 0.405).

Most of the patients were orally or enterally nourished, and intolerance to nutrition was not a problem in the majority of the cases.

Ventilation associated pneumonia (VAP) was the major infectious complication, being present in almost 19% of patients, but there was no association between cholecalciferol supplementation and the presence (c2(2) = 0.206, p = 0.902). or type (c2(10) = 14.530, p = 0.268) of infectious complications during ICU stay.

There were no statistically significant differences between groups with regard to dexamethasone and enoxaparin use.

The median duration of hospitalization was 14 days, with a median of 8 days spent in the ICU, with no statistically significant differences between groups (**Table 5**). A significant number, 29% of patients, died during hospitalization and an additional 30.4% died within the 60 days after clinical discharge. The majority of deaths occurred in the ICU.

**Table 5:** Evolution during hospital stay and patients destiny .

Variable	Total (n=207)	500MU Vitamin D (n= 66)	3MU/day Vitamin D (n= 72)	No Vitamin D supplemented patients (n= 69)	p-value
Hospitalization duration (days): median (IQR) and 95%CI of median ICU 95% CI () Total hospital LOS 95% CI ()	8.0 (10.0) (7–9) 14.1 (13) (14–16)	7.5 (9.2) 95%CI (6–9) 14.5 (12.0) (13-17.5)	8.0 (9.0) 95%CI (5.5-11) 14.0 (15.0) (12-18.5)	8.0(8.0) 95%CI (6–10) 14.0(10.0) (13–17)	0.92 0.97
Destiny – n (%) Discharged Deceased Transferred Rehabilitation ECMO	135 (65.2) 60 (29.0) 5 (2.4) 5 (2.4) 2 (1.0)	47 (71.2) 15 (22.7) 2 (3.0) 1 (1.5) 1 (1.5)	45 (62.5) 24 (33.3) 1 (1.4) 2 (2.8) 0 (0.0)	43 (62.3) 21 (30.4) 2 (2.9) 2 (2.9) 1 (1.5)	0.873
All-cause mortality – n (%) ICU Intrahospitalar total 60 days after clinical discharge	57 (27.5) 60 (29.0) 63 (30.4)	14 (21.2) 15 (22.7) 17 (25.8)	23 (31.9) 24 (33.3) 24 (33.3)	20 (30.0) 21(30.4) 22 (31.9)	0.351 0.873 0.596

IQR, interquartile range; ICU, intensive care unit; LOS, length of stay.

There was no association between 25vitD levels on admission and ICU mortality (p=0.206) or between cholecalciferol supplementation and ICU (H(2) = 0.063, p =0.969) or hospital (H(2) = 0.174, p = 0.917) length of stay, ICU mortality (c2(2) = 2.097, p = 0.351) or hospital mortality (c2(2) = 3.825, p = 0.873).

We assessed age, gender, comorbidities (hypertension, diabetes, heart failure, obesity, BMI, chronic kidney disease, chronic obstructive pulmonary disease), ICU mortality scores (APACHE II, SAPS II), and admission laboratory results (C-reactive protein, procalcitonin, creatinine, albumin) using binomial logistic regression (**Table 6**).

**Table 6:** Binomial logistical regression for mortality outcomes.

ICU mortality		
Variable	OR (95%CI)	p value
**Age**	**OR 1.09 (1.06-1.13)**	**< 0.001**
Gender	OR 1.6 (0.83-3.06)	0.15
**Hypertension**	**OR 5.18 (2.57-11.24)**	**< 0.001**
Diabetes	OR 0.94 (0.47-1.83)	0.89
**Heart failure**	**OR 3.34 (1.14-9.98)**	**0.03**
**Obesity**	**OR 0.46 (0.23-0.87)**	**0.02**
BMI	OR 0.98 (0.92-1.04)	0.48
**Chronic kidney disease**	**OR 3.34 (1.14-9.98)**	**0.03**
**Chronic obstructive pulmonary disease**	**OR 3.34 (1.14-9.98)**	**0.03**
C-reactive protein D1	OR 1.02 (0.98-1.05)	0.29
**Procalcitonin D1**	**OR 1.18 (1.03-1.4)**	**0.03**
**Creatinine D1**	**OR 2.11 (1.29-3.76)**	**0.006**
**Albumin D1**	**OR 0.35(0.15-0.8)**	**0.015**
**APACHE II**	**OR 1.31 (1.2-1.44)**	**< 0.001**
**SAPS II**	**OR 1.11 (1.07-1.16)**	**< 0.001**
**VitD25 D1**	**OR 0.94 (0.89-0.98)**	**0.007**
Intra-hospital mortality		
Variable	OR (95%CI)	p value
**Age**	**OR 1.09 (1.06-1.13)**	**< 0.001**
Gender	OR 1.6 (0.84-3.03)	0.15
**Hypertension**	**OR 5.78 (2.87-12.52)**	**< 0.001**
Diabetes	OR 1.2 (0.61-2.28)	0.59
**Heart failure**	**OR 4.15 (1.43-12.92)**	**0.01**
**Obesity**	**OR 0.51 (0.26-0.96)**	**0.04**
BMI	OR 0.98 (0.92-1.03)	0.45
**Chronic kidney disease**	**OR 4.15 (1.43-12.92)**	**0.01**
**Chronic obstructive pulmonary disease**	**OR 3.08 (1.05-9.2)**	**0.038**
C-reactive protein D1	OR 1.01 (0.98-1.04)	0.42
**Procalcitonin D1**	**OR 1.19 (1.03-1.42)**	**0.029**
**Creatinine D1**	**OR 3.04 (1.71-5.87)**	**< 0.001**
**Albumin D1**	**OR 0.33 (0.14-0.74)**	**0.01**
**APACHE II**	**OR 1.32 (1.22-1.46)**	**< 0.001**
**SAPS II**	**OR 1.11 (1.08-1.2)**	**< 0.001**
**VitD25 D1**	**OR 0.93 (0.88-0.97)**	**0.002**
Alive 60 days after discharge		
Variable	OR (95%CI)	p value
Age	**OR 1.09 (1.06-1.13)**	**< 0.001**
Gender	OR 1.45 (0.76-2.72)	0.26
Hypertension	**OR 5.62 (2.84-11.88)**	**< 0.001**
Diabetes	OR 1.35 (0.7-2.54)	0.36
Heart failure	**OR 3.83 (1.32-11.92)**	**0.015**
Obesity	**OR 0.51(0.27-0.95)**	**0.04**
BMI	OR 0.98 (0.92-1.03)	0.41
Chronic kidney disease	**OR 3.83 (1.32-11.92)**	**0.015**
Chronic obstructive pulmonary disease	OR 2.85 (0.98-8.49)	0.053
C-reactive protein D1	OR 1.01 (0.87-1.04)	0.6
Procalcitonin D1	**OR 1.17 (1.02-1.4)**	**0.039**
Creatinine D1	**OR 2.75 (1.58-5.22)**	**< 0.001**
Albumin D1	**OR 0.37 (0.16-0.82)**	**0.017**
APACHE II	**OR 1.31 (1.21-1.44)**	**< 0.001**
SAPS II	**OR 1.1 (1.06-1.14)**	**< 0.001**
VitD25 D1	**OR 0.93 (0.88-0.97)**	**0.003**

A multivariate analysis adjusting for several independent variables showed no significant effect of different cholecalciferol supplementation strategies on mortality (**Table 7**). Higher vitD25 levels on admission were associated with lower odds of intra-hospital mortality (OR 0.93 with 95%CI (0.09-0.99)).

**Table 7:** Adjusted mortality.

ICU mortality (adjusted for age, hypertension, heart failure, obesity, CKD, COPD, PCT *, Cr *, albumin*, APACHE-II, SAPS-II)		
Variable	Adjusted OR (95%CI)	p value
Vitamin D supplementation with 500M UI	OR 0.69 (0.199 – 2.38)	0.55
Vitamin D supplementation with 3M UI/day	OR 1.23 (0.38-4.08)	0.73
Intra-hospital mortality (adjusted for age, hypertension, heart failure, obesity, CKD, COPD, PCT ^*^, Cr ^*^, albumin^*^, APACHE-II, SAPS-II)		
Variable	Adjusted OR (95%CI)	p value
Vitamin D supplementation with 500M UI	OR 0.67 (0.194 – 2.29)	0.53
Vitamin D supplementation with 3M UI/day	OR 1.17 (0.37-3.83)	0.79
Alive at 60 days after discharge (adjusted for age, hypertension, heart failure, obesity, CKD, COPD, PCT *, Cr *, albumin*, APACHE-II, SAPS-II)		
Variable	Adjusted OR (95%CI)	p value
Vitamin D supplementation with 500M UI	OR 1.09 (0.35-3.44)	0.88
Vitamin D supplementation with 3M UI/day	OR 1.31 (0.44-4.02)	0.63

* evaluated at day 1 of admission.

Supplementation with different cholecalciferol doses did not affect IMV days (**Table 8**), ICU (**Table 9**) or hospital LOS (**Table 10**). (Adjusted R2 = 6.9% for ICU LOS; Adjusted R2 = 1.4% for hospital LOS; Adjusted R2 = 15.0% for IMV).

**Table 8:** Multiple linear regression of numeric/continuous prognostic outcomes for IMV days.

IMV days	Estimate	Std.Error	t value	Pr(<|t|)	Signif.
(Intercept)	-5.4031	10.92995	-0.494	0.621795	
Cholecalc1	0.53197	2.02413	0.263	0.793058	
Cholecalc2	1.10333	2.01802	0.547	0.585374	
Age	-0.16906	0.089277	-1.894	0.06019	.
Gender	-0.77723	1.84479	-0.421	0.674137	
DM	-2.98608	1.93324	-1.545	0.124565	
BMI	0.36027	0.19424	1.855	0.065604	.
COPD	2.89571	2.96698	0.976	0.330657	
PCR	-0.04168	0.10084	-0.413	0.679985	
Hypertension	4.99633	2.17752	2.295	0.023158	*
Obesity	-5.65829	2.18647	-2.588	0.010613	*
CKD	-0.56276	3.45596	-0.163	0.870867	
PCT adm	-0.35053	0.71703	-0.489	0.625656	
Creatinin adm	0.10072	1.86342	0.054	0.956968	
Albumin adm	-0.2125	2.36487	-0.09	0.928521	
APACHE II	0.87739	0.391	3.669	0.000338	***
SAPS II	0.12313	0.09147	1.346	0.180288	
VitD25 adm	0.05614	0.09516	0.59	0.556066	

Signif. Codes: 0(***), 0.001(**), 0.01 (*) 0.05 (.), 0.1 () 1.

Residual sandard error: 10.11 on 149 degrees of freedom (40 observations deleted due to missingness).

Multiple R-sqared: 0.2372, adjusted R-squared: 0.1502.

F-statistic: 2.725 on 17 and 149 DF, p-value: 0.0006054.

**Table 9:** Multiple linear regression of numeric/continuous prognostic outcomes for ICU LOS.

ICU LOS	Estimate	Std.Error	t value	Pr(<|t|)	Signif.
(Intercept)	-1.18341	11.85438	-0.1	0.92061	
Cholecalc1	-0.09788	2.19533	-0.045	0.9645	
Cholecalc2	1.0734	2.1887	0.49	0.62455	
Age	-0.13715	0.09682	-1.417	0.15869	
Gender	-1.55395	2.00082	-0.777	0.43859	
DM	-1.86937	2.09675	-0.892	0.37407	.
BMI	0.41029	0.21067	1.948	0.05335	
COPD	3.65912	3.21793	1.137	0.25732	
PCR	-0.10233	0.10937	-0.936	0.351	.
Hypertension	3.94329	2.36169	1.67	0.09708	**
Obesity	-6.46701	2.3714	-2.727	0.00716	
CKD	0.25738	3.74826	0.069	0.94535	
PCT adm	-0.27828	0.77768	-0.358	0.72097	
Creatinin adm	0.86204	2.02103	0.427	0.67033	
Albumin adm	-0.38818	2.56489	-0.151	0.87991	*
APACHE II	0.57632	0.25933	2.222	0.02776	
SAPS II	0.12119	0.09921	1.222	0.22377	
VitD25 adm	0.1387	0.1032	1.344	0.181	

Signif. Codes: 0(***), 0.001(**), 0.01 (*) 0.05 (.), 0.1 () 1.

Residual sandard error: 10.96 on 149 degrees of freedom (40 observations deleted due to missingness).

Multiple R-sqared: 0.164, adjusted R-squared: 0.06862.

F-statistic: 1.719 on 17 and 149 DF, p-value: 0.04493.

**Table 10:** Multiple linear regression of numeric/continuous prognostic outcomes for Hospital LOS.

Hospital LOS	Estimate	Std.Error	t value	Pr(<|t|)	Signif.
(Intercept)	22.68096	16.122	1.407	0.1616	
Cholecalc1	0.85938	2.98565	0.288	0.7739	
Cholecalc2	2.29877	2.97663	0.772	0.4412	
Age	-0.01499	0.13167	-0.114	0.9095	
Gender	-2.60799	2.72112	-0.958	0.3394	
DM	-1.77694	2.85159	-0.623	0.5341	
BMI	0.35704	0.28651	1.246	0.2147	
COPD	-0.45433	4.37639	-0.104	0.9175	
PCR	-0.17533	0.14875	-1.179	0.2404	
Hypertension	4.91991	3.21191	1.532	0.1277	
Obesity	-4.20553	3.22511	-1.304	0.1942	
CKD	-7.48185	5.09765	-1.468	0.1443	
PCT adm	-0.54261	1.05764	-0.513	0.6087	
Creatinin adm	2.78817	2.7486	1.014	0.312	
Albumin adm	-5.79827	3.48826	-1.66	0.0991	.
APACHE II	0.14045	0.35268	0.398	0.691	
SAPS II	0.01584	0.13492	0.117	0.9067	
VitD25 adm	0.24046	0.14036	1.713	0.0888	.

Signif. Codes: 0(***), 0.001(**), 0.01 (*) 0.05 (.), 0.1 () 1.

Residual sandard error: 10.11 on 149 degrees of freedom (40 observations deleted due to missingness).

Multiple R-sqared: 0.2372, adjusted R-squared: 0.1502.

F-statistic: 1.141 on 17 and 149 DF, p-value: 0.3212.

**Figure 1:**
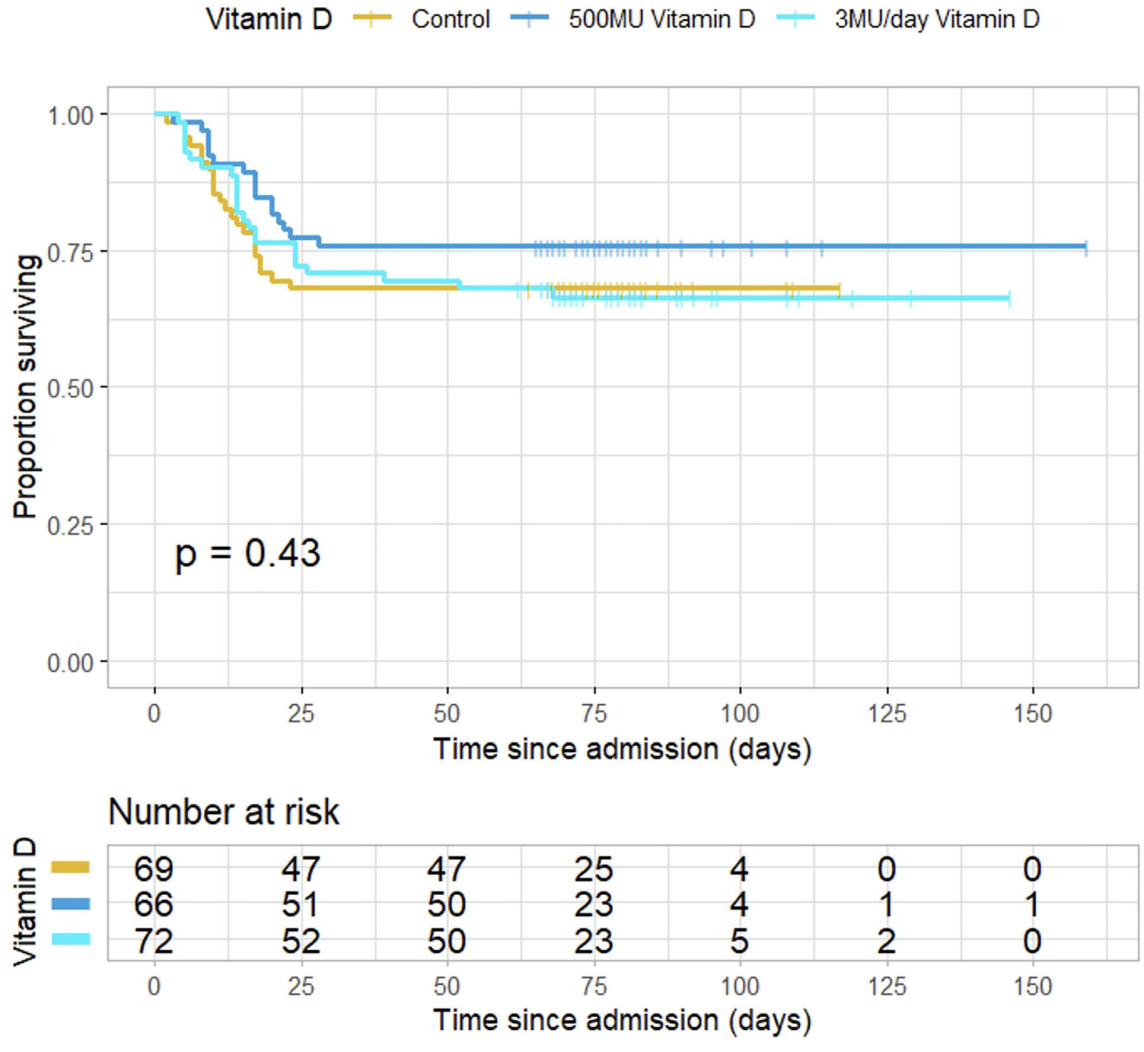
Survival of patients in the three groups of patients. HR = 1.012 95%CI (0.75-1.37).

A survival curve is presented, HR = 1.012 (**Figure 1**).

## 4 Discussion

Despite the widely known immunomodulatory and antimicrobial effects of vitD *in vitro* and health benefits, our study did not demonstrate any change in the clinical course and prognosis of patients admitted to the intensive care unit. However, patients admitted in the ICU, who presented with higher levels of vitD25 had lower odds of hospital mortality.

Covid-19 pandemic provided a unique opportunity to gather a homogeneous population of critically ill patients, with severe SARS-CoV-2 pneumonia (all patients were admitted to a single intensive care unit with a similar medical approach and therapy in accordance with global indications, reducing bias possibility).

The sample size (n=207) was quite adequate and with similar patient characteristics in all the three groups. Mortality scoring indices were comparable between groups, with a relatively low risk. These findings are in line with a common characteristic of these patients mainly admitted with severe lung disease and absence of other organ failure.

It should be noted that patients enrolled in this study were rigorously monitored clinically and laboratory, despite the lack of statistically significant results. The use of vitD as a supplement, even in high doses, proved to be safe, with no adverse effects.

This study reinforces our idea that there is still a lot to know regarding vitD targets and its physiologic effects.

The group of patients supplemented with 500MU in the first 48h showed a significant decrease in the number of failing organs on the third day but not on the seventh. Could this indicate the need for a higher dose or longer period of administration?

In the group not supplemented there was a significant negative relation between organ failures and 25vitD levels on the seventh day, but not on admission or on the third day. This may be related to the continuous decrease in 25vitD levels, suggesting that lower levels are harmful. It is possible that vitD status previous to ICU admission has a protective effect and perhaps, because of that there were not significant differences between groups, since although not significant, the group of patients not supplemented had a higher basal level of 25vitD on admission.

Although we could not demonstrate a beneficial effect of vitD supplementation on prognosis of critically ill patients admitted to the ICU with severe SARS-CoV-2 pneumonia, we cannot exclude potential benefits on mortality either.

In fact, initial higher serum vitD was associated with less organ failures, and higher initial supplementation seemed to have a similar effect. VitD effects may be slow to manifest, since supplemented cholecalciferol needs to be metabolized by hepatic and renal hydroxylation, and subsequently active vitD acts mainly through the relatively slow pathways of steroid hormones.

In critically ill patients these metabolic pathways can be slowed or compromised (68). It may also occur that because of critical illness, patients may need higher vitamin D doses or for a longer period of time. Another aspect to take into account is that there may exist vitamin D binding protein polymorphism contributing, with acute illness, to a state of cholecalciferol resistance, since high doses of supplementation do not appear to change clinical course or prognosis of patients. During Covid-19 disease a study including 491 patients was developed and found that Portuguese population had a vitamin D binding protein polymorphism that might explain the lower vitD levels and the higher severity of the disease (69), what may also justify the need for a greater supplemental intake of vitamin D.

Therefore, it is plausible that high doses beginning in the first days and given during a large period may be more effective, even more in populations with vitamin D binding proteins polymorphisms.

Maintaining elevated baseline levels of 25vitD to the onset of disease could be crucial. Elevated vitamin D targets in the general population have been associated to improved health, particularly protection against infections and immunological responses. Should the general population be supplemented to attain higher physiological concentrations? If supported by evidence, the anticipated health benefits from widespread supplementation are expected to greatly outweigh the associated costs. This would imply a comprehensive public health strategy on a global scale.

## Data Availability

The raw data supporting the conclusions of this article will be made available by the authors, without undue reservation.
